# Evaluation of Shear Bond Strength of Resin-Based Composites to Biodentine with Three Types of Seventh-Generation Bonding Agents: An *In Vitro* Study

**DOI:** 10.1155/2022/2830299

**Published:** 2022-07-30

**Authors:** Huda Abbas Abdullah, Zahraa Abdulaali Al-Ibraheemi, Zanbaq Azeez Hanoon, Julfikar Haider

**Affiliations:** ^1^Department of Conservative Dentistry, College of Dentistry, Tikrit University, Tikrit, Iraq; ^2^Department of Conservative Dentistry, Faculty of Dentistry, University of Kufa, Najaf, Iraq; ^3^Department of Engineering, Manchester Metropolitan University, Manchester M1 5GD, UK

## Abstract

**Methods:**

Forty-eight acrylic blocks having central holes with a nominal diameter of 4 mm and a depth of 2 mm were prepared. The holes of the acrylic blocks were filled with Biodentine, which was prepared following the guidelines provided by the manufacturer. Then, the specimens were divided into six groups (*n* = 8). Groups 1, 2, and 3, Tetric N-Ceram composite bonded to Biodentine with Tetric N-bond, Xeno V+, Bond Force bond, respectively. Group 4, 5, and 6, Filtek Z350 bonded to Biodentine with the same three adhesives. The specimens were placed in distilled water for 24 hours and tested for the SBS in a universal testing machine at a crosshead speed of 1 mm/min. The test data were listed in a table and independent samples *t*-test and analysis of variance (ANOVA) were conducted as a part of the statistical analysis.

**Results:**

The Tetric N bonding agent achieved the highest SBS followed by Bond Force, and Xeno V and highly significant difference was found. On the other hand, an overall increase in the SBS values of the Tetric N-Ceram resin was noticed in comparison with the Filtek Z350 and the differences was statistically significant. Although the specimens failed in adhesive, cohesive and mixed fracture modes but the cohesive was found to be the dominant failure mode in all groups.

**Conclusion:**

Among the tested bonding agents and resin composites, the Tetric N-Ceram composite bonded by Tetric N-bond self-etch adhesive with the Biodentine showed the highest SBS compared to the other combinations.

## 1. Introduction

Biodentine was presented as a dentine coating material that is used in deep cavities to reduce the bulk of filling material and protect the pulp. It is recommended for using it underneath restorative resin composites and a repair material for endodontic applications due to its favorable biocompatible, bioactive, and biomineralizing characteristics [1, 2]. Tricalcium silicate accompanied with calcium carbonate and zirconium oxide is the chief powder element of Biodentine [3]. The liquid component consists of calcium chloride solution with a reducing agent, which is responsible for gaining certain advantages such as a short setting period and a high compressive strength value comparable to the natural dentine [4].

Biodentine can infiltrate through the dentinal tubules and show ability to crystallize while adhering with dentine, thereby improving bonding [5]. The adhesion between Biodentine and restorative material is important for the success of final restoration. In the case of adhesive failure, insufficient adhesion of the materials at the boundary of the restoration causes an internal gap, which permits infiltration of microorganisms through the gap, leading to complications such as sensitivity, secondary caries, microleakage, infection, discoloration, and finally the restoration failure [6, 7].

Evolution of the dental adhesive systems can be identified in terms of elemental composition in the materials with their internal chemistry, application procedure, and clinical performance [8–10]. 7^th^generation bonding agents are now available in the market and these are also known as single-solution or all-in-one products. It is important to gain further understanding about performance of the products in terms of accomplishing etching, priming, and bonding/sealing using a single solution. Furthermore, it is necessary to evaluate the bonding efficacy of these newer simplified bonding agents to provide some guidance to the dentists and dental technicians for selecting the best alternative adhesive material from a large pool of bonding materials available commercially. Bonding quality can be enhanced by selecting suitable combinations of dental adhesives, application techniques, and curing strategies.

Resin composites have been the material of choice for esthetic restorations in anterior and posterior teeth due to the advances made in their chemical, physical, and mechanical properties. Despite their improved characteristics, the composites have an intrinsic characteristic of shrinkage during the curing process as monomers transform from free floating molecules to rigid polymeric chains [11]. This contraction produces tensile stress that tends to concentrate at the tooth/restoration interfaces, weakening the adhesive union and creating marginal gaps that can lead to microleakage, postoperative sensitivity, and recurrent caries [12, 13]. The presence of inorganic filler particles within the resin composites has significantly reduced the polymerization shrinkage. The particle size and shape, as well as the organic-resin matrix have all been improved [14] over the past years and nanotechnology particularly has assisted the development of inorganic fillers for the newer composite materials [14, 15]. Nanoparticle-filled resins, which contain nanoparticles and nanoclusters, and nanohybrid resins, which combine nanofillers with small particles, are the two forms of nanotechnology-based composites available now [16]. An optimal distribution of different sized particles allows the inorganic content of the composite resin to be increased, resulting in a potential reduction in polymerization shrinkage [14].

Limited information is available in the literature on the bond strength between the resin composites and the Biodentine with different boning agents; hence, their bonding mechanism is not fully understood. The study aimed to determine the SBS between resin-based composites and Biodentine using three types of 7th generation bonding agents.

The null hypotheses stated that (1) no significant differences would be present in terms of SBS between the three adhesive systems for a particular resin composite and (2) no significant differences would be present between the two resin composites for a particular adhesive system.

## 2. Materials and Methods

### 2.1. Sample Preparation

**Figure 1:**
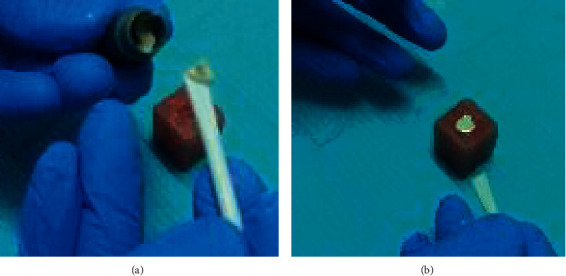
Application of mixed Biodentine: (a) acrylic block with cylindrical cavity and (b) filling cavity with Biodentine.

Forty-eight acrylic blocks having central holes with a nominal diameter of 4 mm and a depth of 2 mm were prepared. Biodentine were prepared following the manufacturer's guidelines and placed in the hole of each acrylic block as shown in Figure 1.

The specimens were kept at 37°C with 100% humidity for 24 hours to facilitate setting. Two types of composites: nanohybrids resin, Tetric-N ceram, and nanofilled resins, FiltekZ350 and three commercially available types of seventh generation bonding agents, Tetric N-bond, Xeno V+, and Bond Force were tested in this study. The adhesive systems and the resin composites considered in this study and their chemical compositions are presented in Table 1.

The specimens were separated into six groups (*n* = 8) based on different combinations of resin composites and adhesives (Table 2).

**Figure 2:**
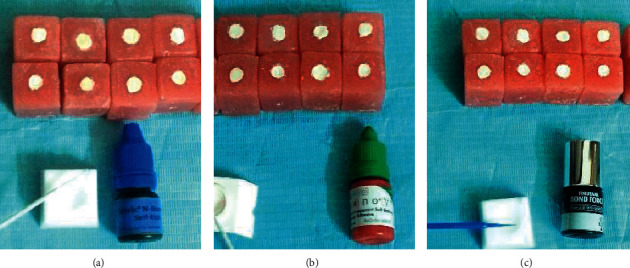
Holes in the acrylic block filled with the Biodentine for different bonding agents: (a) Tetric N-bond (b) Xeno (V) and (c) Bond Force.

The corresponding bonding agents are applied to Biodentine according to manufacturer instructions as shown in Figure 2. The restorative resin materials are then applied as a 2-layer increment into a transparent cylindrical shaped plastic molds with an internal diameter of 2 mm and a height of 3 mm.

**Figure 3:**
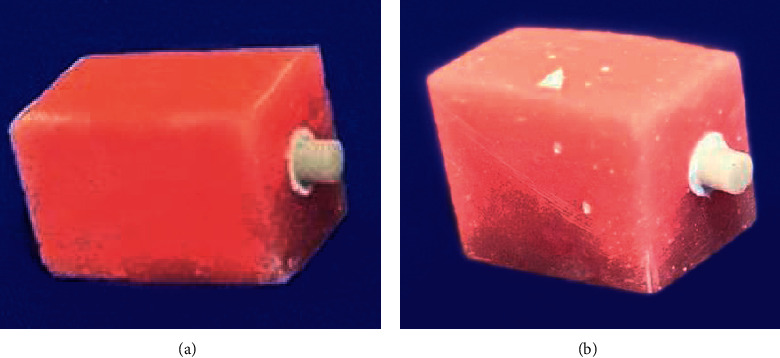
SBS test samples: (a) Tetric-N Ceram and (b) FiltikZ350.

Polymerization was completed by light curing for 40 seconds in a curing unit (Perfection Plus, UK; light intensity: 800 MW/cm^2^). Each composite cylinder was also cured for an additional 40 seconds after removal from the mold.  Figure 3 presents the complete test sample after attaching the resin composites with the Biodentine. All specimens were kept in distilled water for 24 hours prior to SBS testing to mimic the oral environment. All sample preparation was made by a single operator by following the standardized procedure to avoid any inconsistencies in the prepared samples.

### 2.2. Shear Bond Strength (SBS) Test

**Figure 4:**
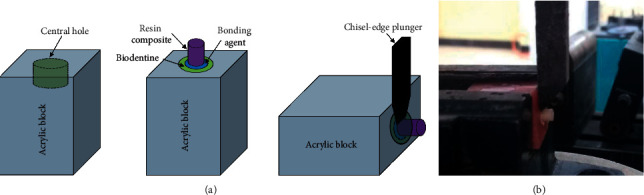
(a) Schematic diagram of the sample preparation with setup for shear bond strength test and (b) image of actual test setup.

Shear bond strength is a commonly used laboratory test to assess the bonding performance of dental adhesive systems [17]. Shearing action is present in the posterior teeth during chewing, and therefore, the SBS test would represent the performance assessment of the restorative material under clinical condition. During the SBS test, each specimen was placed in a universal testing machine (Hoytom machine), and force was applied by the machine on each specimen at a crosshead speed of 1 mm/min. Achisel-edge plunger was secured onto a vertically movable crosshead in the testing machine and adjusted its position to ensure that the leading edge was aimed at the Biodentine/adhesive interface (Figure 4) force was continuously applied until the failure took place.

The force required to debond the restorative material was recorded in Newton (N) and the bond strength *σ* in MPa was determined by dividing the shear force (F) in N by the adhesion area A.

(1) $$\begin{array}{c} \sigma =\frac{F}{A}=\frac{F}{\pi r^{2}}, \end{array}$$

where *r* is the radius of the central hole.

### 2.3. Failure Mode Identification

The failure modes were identified under a low magnification (×25) light microscope to assess the performance of different combinations of adhesive and resin composite systems. Three failure modes were assessed: pure adhesive, cohesive within Biodentine or resin composite, and mixed failure with adhesive and cohesive types. Fracture behavior was analyzed by an independent researcher who was completely unaware about the experimental groups during sample preparation to avoid any bias.

### 2.4. Statistical Analysis

The test data were listed in a table and independent samples *t*-test and analysis of variance (ANOVA) were conducted as a part of the statistical analysis using IBM SPSS 21 software. A two-way ANOVA was used to determine the effects of the adhesive systems and restorative materials on the SBS and their interactions with a significance level set at *P* ≤ 0.05.

## 3. Results

**Figure 5:**
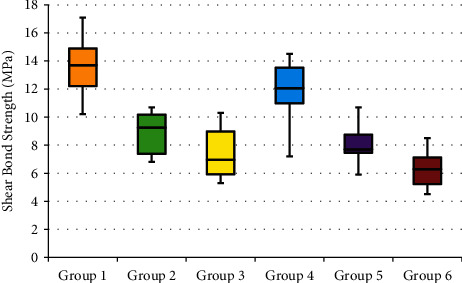
Box plot representing the shear bond strength (SBS) of the study groups.

The mean values and standard deviations of SBS for all groups are shown in Table 3 and Figure 5.

The results clearly demonstrated that the highest mean SBS was recorded for Group 1, in which Tetric N-Ceram composite was bonded to the Biodentine with Tetric N bond (13.59 MPa), while the lowest mean SBS was recorded for Group 6, in which FiltekZ350 composite was bonded to the Biodentine with Xeno V (6.33 MPa).

On the other hand, there was an overall increase in the SBS values of Group 1, Group 2, and Group 3 that Tetric N-Ceram resin composite bonded with the three different bonding agents in comparison with Group 4, Group 5, and Group 6 in which the FiltekZ350 resin composite was used and the difference was statistically significant (Table 4). The Tetric N bonding agent represented the highest value of SBS was achieved followed by Bond Force and Xeno V, respectively, and the difference was highly significant. Although the resin composites and bonding agents caused a statistically significant difference, the interaction of these two did not make a significant difference.

**Figure 6:**
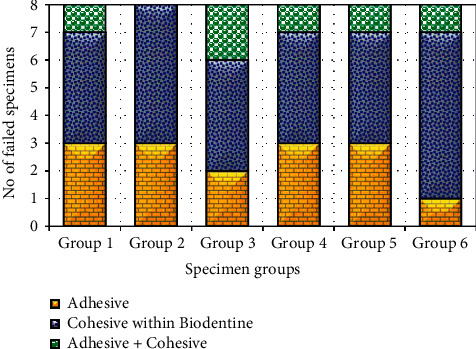
Distribution of failed specimens during SBS tests.

**Figure 7:**
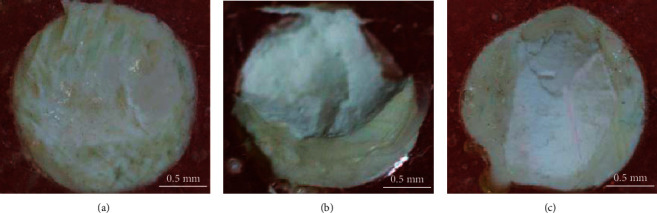
Representative failure modes in the specimens: (a) adhesive, (b) cohesive within Biodentine, and (c) adhesive + cohesive.

Figure 6 presents modes of failures in different specimen groups. It was clear that cohesive failure within Biodentine was dominant (50% to 75%) among all the groups followed by adhesive and mixed type (adhesive + cohesive) failures. In this case, 38% adhesive failure was recorded in Group 1, Group 2, Group 4, and Group 5 whereas 25% adhesive failures were found in Group 3 and only 12% in Group 6 failed adhesively. On the other hand, the mixed type of failure in al groups was found within a range between 0% and 25%.  Figure 7 shows examples of different failure types observed in the specimens.

## 4. Discussion

The most critical factors that determine the clinical success of a dental restoration could be the bonding strength between the restorative resin materials and the enamel or dentin, as well as the strength between the restorative material and the cavity liner. In this study, the SBS of two resin composites (Tetric N-Ceram and Filtek Z350 XT) and Biodentine with three 7^th^ generation adhesive systems (Tetric N, Xeno V, and Bond force) was evaluated. To the author's best knowledge, no studies were reported on the SBS of the combination of resin composites and adhesives considered here. For each of the resin composites, significant differences in SBS were found among the three bonding agents studied here. Therefore, the first null hypothesis was rejected. Again, for each of the bonding agent, the resin composites showed significant difference in bond strength. Thus, the second hypothesis was also rejected.

In this study, the mean SBS values ranged between 6.33 and 13.59 MPa, which was lower than the range of bond strength (17–20 MPa) recommended for producing strong restoration without any gap at the boundary [18]. The low SBS of the Biodentine seemed to be owing to the low initial strength of the material and other studies also argued the same [19, 20]. Biodentine being a porous material requires minimum two weeks to fully crystallize and to reach the required bulk strength, which can resist the stress caused by polymerization [20, 21]. In the present study, the bonding was applied to the Biodentine after 24 hours. This could explain the relatively low bond strength.

Since there was no indication of the resin structure attached to the Biodentine, this indicated that the bond created with the composite resins might be purely micromechanical [22]. In addition, modification of the surface physical aspects of by surface treatment can influence the micromechanical bonding [23]. Choi et al. conducted experiments with the Filtek Z250 resin composite bonded to a human teeth dentin surface by three universal adhesive systems and varying air-drying times of 0, 5, and 10 s [24]. It was concluded that the bond strength was affected by wetness of the dentin surface.

The SBS results found in this study was higher than the findings of Altunsoy et al. (1.69 MPa) [23] and Deepa et al. (5.66 MPa) [19], comparable with that of Krawczyk-Stuss et al. (6.2 MPa) [25] and Shin et al. (6.87) [26] and lower than that reported by Odabaş et al. [27] (11.057–15.193 MPa). This could be due to the difference in adhesive systems, adhesive strategies, resin materials, experimental test set-up, sample preparation, or operator variable.

Based on the SBS results obtained in this study, there were statistically significant differences between Tetric N-ceram and FiltekZ350 XT, with the former showing higher bond strength values. The variation in composition of the two resin composites along with the nanohybrid structure in Tetric N-Ceram might explain the difference in the SBS. Furthermore, high concentrations of TEGDMA diluent monomer in the FiltekZ350XT might have increased its shrinkage, therefore decreasing the bond strength since Kim et al. reported high volumetric shrinkage with Z350XT when compared to several bulk-fill resin-based composites (RBCs) [28]. Govindaraju et al. found that Filtek Z350XT showed the least SBS and it was statistically significant when compared to other two materials (Dentsply Ceram X and GC Solare Sculpt) [29]. However, owing to the limited information available from the manufacturers on the exact composition of the resin composites or the bonding agents used in this study, the difference in bonding strength could not be fully explained.

Mean SBS values of all groups were ranked as follows: Group 1 > Group 4 > Group 2 > Group 5 > Group 3 > Group 6. Based on the results of six combinations of bonding agents and restorative materials, Tetric N-bond self-etch and Tetric N-Ceram composite (Group 1) was recommended for obtaining the strongest bond ensuring the long-term clinical success.

The 7^th^generation bonding agents are easy to handle and apply. The traditional steps of bonding process such as etching, priming, and bonding agent application can be carried out in one step leading to a significant reduction in the application time and improve consistency and quality of the restoration with fewer errors related to technical procedures. Therefore, the bonding system was expected to show lower failure rates in clinical applications.

The 7^th^generation bonding agents can be categorized as strong (pH of smaller than 1), intermediate strong (pH of approximately 1.5), and mild self-adhesives (pH of approximately 2.0) [30]. Bond Force has a pH of 2.3, Xeno V has <2, and Tetric N-bond self-etch is 1.5 Therefore, the Tetric N-self-etch is an intermediate strong adhesive while the Bond Force and Xeno V are a mild self-adhesive. Mild self-adhesives have a comparatively weaker bond potential [30]. This may be a reason for statistically highly significant differences in bonding strength between the bonding agents in this study. In contradiction to this study, Nikhil et al. [31] concluded that the mild self-etch adhesives appeared the most promising especially with regards to the bond stability, and Jamadar et al. found the pH values did not influence the SBS [32]. The structural differences among the bonding agents in terms of chemical content might cause differences in degree of polymerization or polymerization shrinkage at the interface, cross-linking, and depth of penetration in composite or Biodentine [33]. These factors could have led to the difference in bonding strength.

In general, adhesive failure indicates poor bonding between the resin, Biodentine, and adhesive but stronger bonding was defined by cohesive failure [34, 35]. In this study, no clear trend between the failure modes and the SBSs of different groups were noticed. The cohesive failure within the Biodentine could be due to lower bulk compressive strength of the Biodentine used here [21]. Although many studies reported about the SBS between resin composites and Biodentine but only a few studies reported about the failure modes. Altunsoy et al. [23] studied the SBS between different combinations two flowable composites and three pulp capping materials: mineral trioxide aggregate (MTA), Biodentine, and calcium-enriched mixture (CEM). Cohesive or mixed failures were found with the mixed type being the dominant mode and without any adhesive failure, which was in contradiction to this study where all three failure types were present and the cohesive type was more dominant than the others. Tulumbaci et al. [34] assessed SBS of three resins (composite, compomer, and resin-modified glass ionomer) with MTA and Biodentine using Prime and Bond NT adhesives. Cohesive failure was dominant in the MTA groups but the groups with Biodentine showed mainly the adhesive failures, which were clearly linked with the SBS values. Similarly cohesive and adhesive failures were also found by Carretero et al. [18] when the SBS between Biodentine and a composite resin with different adhesives were tested. However, Deepa et al. [19] and Raina et al. [35] observed three types of failures while a resin composite was bonded to different materials including Biodentine. Cohesive failure within Biodentine found in the studies also supported the observation in this study. Adhesive failures in different groups recorded here indicated lack of strong chemical bonding formed either between adhesives and Biodentine or adhesives and resin composites [21].

The SBS tests were carried out after storing in distilled water for only 24 hours. However, conducting SBS tests after subjecting the bonding systems to different physical, mechanical, or thermal aging [17, 36, 37] or any contamination in the bonding system such as saliva or blood [38] can represent a situation close to the clinical condition. This would be considered in our future studies. This study was carried out to assess the performance of the bonding systems *in vitro* in order to ensure experiments conducted under controlled conditions, which is a standard technique commonly accepted by the dentistry research community. However, this experimental condition might not accurately replicate the clinical situation *in vivo*.

In this study, only 24 hours was permitted to set the Biodentine with resin composites and this might be responsible for the overall poor strength lower than the minimum strength requirement. Carretero et al. found improved adhesion between nanohybrid composite and Biodentine at 24 h when compared to a shorter setting time (12 min). Therefore, it was suggested to consider longer setting time for Biodentine [18]. Future studies could be carried out to evaluate the influence of longer setting time on the SBS with different combinations of adhesive and restorative systems.

### 4.1. Clinical Significance

The results of this study offered a recommendation for the dentists and clinicians to select a specific bonding system for dental restoration in order to ensure long-term clinical performance and patient's satisfaction.

## 5. Conclusion

Within the limitations of the study, it was concluded that among the three commercially available 7^th^ generation bonding agents (i.e., Tetric N-Bond, Xeno V, and Bond Force) used for bonding two esthetic resin composite materials (Tetric-N Ceram, nanohybrid, and Filtek Z350XT) to Biodentine, the combination of Tetric N-Bond with Tetric-N Ceram has been recommended due to the highest SBS. A reliable bonding system can be beneficial when used for esthetic purpose leading to a long-term clinical success of the restoration due to the improved restoration stability.

## Figures and Tables

**Table 1:** List of materials used with their manufacturers and application procedures.

Materials	Composition	Manufacturer	Steps of application
Biodentine tricalcium silicate cement	Powder: tricalcium silicate, dicalcium silicate, calcium carbonate Zirconium oxide (radio opacifier) Iron oxide (colouring agent) Liquid: calcium chloride, accelerator, hydrosoluble, polymer, water reducing agent	Setodent, France	Five doses liquid and powder supplied for 30 s with a mixed amalgamator

Filtek Z350XT	Organic phase: UDMA, Bis-GMA, Bis-EMA, TEGDMA Inorganic phase: silica (20 nm non-agglomerated/aggregated), zirconia (4–11 nm non-agglomerated/aggregated and agglomerated), clusters, zirconia/silica aggregated particles (20 nm silica particles combined with 4–11 nm zirconia) Filler content (vol.%) 63.3%	3M, ESPE, USA	Apply increments of 2 mm and light-cure for 20 s

Tetric N-Ceram nano-hybrid	Organic phase: dimethacrylates TEGDMA Inorganic phase: barium aluminum silicate glass (0.4–0.7 *μ*m), ytterbium trifluoride (200 nm), mixed oxides (160 nm) and copolymers 80–81 barium glass filler, ytterbium trifluoride, mixed oxide (0.04–3.0 nm) Bis-GMAb, Bsis-EMA	IvoclarVivadent, Liechtenstein	Apply increments of 2 mm and light-cure for 20 s

Tetric N-bond self-etch	Bis-acrylamide derivative, bis-methacrylamide dihydrogenphosphate, amino acid acrylamide, hydroxyalkyl metharylamide, nano-filler, water, stabilizers	IvoclarVivadent, Liechtenstein	Apply bonding agent and rub for 20 seconds air dry for 5 sec Light cure for 10 sec

Xeno V+	Bifunctional acrylate, acidic acrylate, functionalized phosphoric acid ester, water, tertiary butanol, initiator, stabilizer.	Dentsply, Germany	Apply it sufficiently pooling then gently agitate the adhesive for 20 sec. Evaporate solvent thoroughly and cure for 10 seconds

Bond force	Phosphoric acid Monomer, Bisphenol a di (2-hydroxypropoxy) dimethacrylate (Bis-GMA), triethylene glycol dimethacrylate, HEMA, camphorquinone, alcohol, water.	Tokuyama dental Tokyo, Japan	Apply bonding agent and rub for 20 seconds Weak air dry for 5 sec. Strong air dry for 5+ sec. And light cure for 10 sec

**Table 2:** Experimental groups with two resin composites and three adhesives.

Groups	Resin composites	Adhesives
Group 1	Tetric N-Ceram composite	Tetric N-bond self-etch
Group 2	Tetric N-Ceram composite	Xeno V+
Group 3	Tetric N-Ceram composite	Bond force
Group 4	Filtek Z350XT	Tetric N-bond self-etch
Group 5	Filtek Z350XT	Xeno V+
Group 6	Filtek Z350XT	Bond Force

**Table 3:** Mean values of SBS for different combinations of restoration materials and bonding agents. ^a–f^: same superscript letters within a row suggest nonsignificant statistical difference (*P* < 0.05).

Labels	Group 1	Group 2	Group 3	Group 4	Group 5	Group 6
Mean	13.59^a^	7.44^b^	8.9^c^	11.78^d^	6.33^e^	8.13^f^
Std deviation	2.061	1.819	1.431	2.31	1.389	1.41
Min	10.20	6.80	5.30	7.20	5.90	4.50
Q1	12.20	7.38	5.93	10.98	7.45	5.23
Median	13.70	9.25	6.95	12.05	7.70	6.30
Q3	14.90	10.18	8.98	13.53	8.75	7.13
Max	17.10	10.70	10.30	14.50	10.70	8.50
IQR	2.70	2.80	3.05	2.55	1.30	1.90

Q1-quartile 1, Q2-quartile 2, IQR: inter quartile range.

**Table 4:** Two-way ANOVA analysis.

Variation source	Type III sum of squares	df	Mean square	*F*	Sig
Corrected model	306.784^a^	5	61.357	17.078	0.000 (HS)
Intercept	4203.763	1	4203.763	1170.092	0.000 (HS)
Restorative material	18.253	1	18.253	5.081	0.029 (S)
Bond agent	286.290	2	143.145	39.844	0.000 (HS)
Restoration^*∗*^bond	2.240	2	1.120	0.312	0.734 (NS)
Error	150.893	42	3.593		
Total	4661.440	48			
Corrected total	457.677	47			

a. *R* squared = 0.670 (adjusted *R* squared = 0.631); ^*∗*^Relationship or the differences between resin composites and bonding agents.

## Data Availability

The data used to support the findings of this study are included within the article.
